# First Event-Related Potentials Evidence of Auditory Morphosyntactic Processing in a Subject-Object-Verb Nominative-Accusative Language (Farsi)

**DOI:** 10.3389/fpsyg.2021.698165

**Published:** 2021-12-16

**Authors:** Simin Meykadeh, Arsalan Golfam, Ali Motie Nasrabadi, Hayat Ameri, Werner Sommer

**Affiliations:** ^1^Department of Linguistics, Tarbiat Modares University, Tehran, Iran; ^2^Institut für Psychologie, Humboldt-Universität zu Berlin, Berlin, Germany; ^3^Department of Biomedical Engineering, Shahed University, Tehran, Iran; ^4^Research Center of Persian Language and Literature, Tarbiat Modares University, Tehran, Iran; ^5^Department of Psychology, Zhejiang Normal University, Jinhua, China

**Keywords:** Farsi, N100, P600, formant, SOV word order, sentence wrap-up, morphosyntactic violations, nominative-accusative language

## Abstract

While most studies on neural signals of online language processing have focused on a few—usually western—subject-verb-object (SVO) languages, corresponding knowledge on subject-object-verb (SOV) languages is scarce. Here we studied Farsi, a language with canonical SOV word order. Because we were interested in the consequences of second-language acquisition, we compared monolingual native Farsi speakers and equally proficient bilinguals who had learned Farsi only after entering primary school. We analyzed event-related potentials (ERPs) to correct and morphosyntactically incorrect sentence-final syllables in a sentence correctness judgment task. Incorrect syllables elicited a late posterior positivity at 500–700 ms after the final syllable, resembling the P600 component, as previously observed for syntactic violations at sentence-middle positions in SVO languages. There was no sign of a left anterior negativity (LAN) preceding the P600. Additionally, we provide evidence for a real-time discrimination of phonological categories associated with morphosyntactic manipulations (between 35 and 135 ms), manifesting the instantaneous neural response to unexpected perturbations. The L2 Farsi speakers were indistinguishable from L1 speakers in terms of performance and neural signals of syntactic violations, indicating that exposure to a second language at school entry may results in native-like performance and neural correlates. In nonnative (but not native) speakers verbal working memory capacity correlated with the late posterior positivity and performance accuracy. Hence, this first ERP study of morphosyntactic violations in a spoken SOV nominative-accusative language demonstrates ERP effects in response to morphosyntactic violations and the involvement of executive functions in non-native speakers in computations of subject-verb agreement.

## Introduction

Grammatical features of languages are highly diverse and show distinct neural underpinnings. ERPs provide information about online language processing at high time resolution and consistently show two signals of syntactic sentence processing, the left anterior negativity (LAN; e.g., Friederici et al., 1993) between 300 and 400 ms, and the subsequent P600, a posterior positivity between 500 and 700 ms (Dube et al., 2016; for reviews Kuperberg, 2007; Leckey and Federmeier, 2019). These components are similar in written and spoken sentences but often start earlier in auditory presentation (Hasting and Kotz, 2008; Shen et al., 2013). LAN and P600 have been associated with morphosyntactic error detection and reanalysis processes, respectively (Friederici, 2002). The P600 was also proposed to reflect syntactic integration difficulty, operationally defined as energy required to reactivate previous predictions and integrate them with current input (Kaan et al., 2000).

LAN and P600 have mainly been observed in languages with SVO or flexible word order (e.g., English, Dutch, Spanish; German). Irrespective of stimulus modality, some studies did not find a LAN; however, subject-verb agreement violations consistently elicit P600 effects, albeit with different amplitudes, latencies and scalp distributions. Therefore, morphosyntactic processing is closely associated with the P600. Conversely, subject-verb agreement manipulations often induced a LAN followed by a P600. Molinaro et al. (2011) reviewed evidence supporting the widely accepted idea that the LAN represents basic syntactic processes focused on morphophonological cues.

ERP studies on subject-verb agreement in SOV languages have either recruited languages with ergative-absolutive (Basque) or split-ergative (Hindi) patterns. Although Hindi has a rich verb agreement morphology and canonical verb final word order, Nevins et al. (2007) placed the critical verb inside sentence-initial adverbial clauses, to avoid sentence-final positions. Using nominative-marked “subjects,” agreement violations elicited a P600 effect (around 600–1,000 ms) but no LAN. In contrast, Choudhary et al. (2009) found a negativity for violations in both, perfective (ergative-marked case) and imperfective (nominative-marked case) sentences, followed by a parietal positivity only in ergative-marked violations. Using Basque, subject-verb agreement violations in transitive sentences elicited posterior negativities, followed by a P600 (Zawiszewski and Friederici, 2009; Zawiszewski et al., 2016) or a P600 without negativity (Dıaz et al., 2011). Chow et al. (2018) observed a P600 in both transitive and intransitive sentences, but an early posterior negativity was seen only in intransitive violations, which was interpreted as reflecting distinct neurocognitive mechanisms for processing agreement with transitive (ergative-marked) versus intransitive subjects (absolutive-marked). Hence, verbal agreement may have language-specific rather universal ERP correlates. The present study will explore, the neural correlates of syntax processing in Farsi, an SOV language with typologically nominative-accusative pattern.

We manipulated subject-verb agreements in spoken Farsi, where all morphosyntactic information of verbs are encoded in the suffix. We time-locked ERPs to target syllable onset to optimally capture syntax violation effects. In subject-verb agreement, verb inflection matches in person, number or gender with a core argument of the verb (Baker, 2008). In some languages, like Japanese, the inflectional patterns of verbs are not affected by person, number or gender while in others, such as Farsi, or Turkish, verbs have six grammatical persons and are inflected for three singular and three plural persons.

Neurocognitive models of auditory sentence processing offer different accounts for the interplay of syntax and semantics. Serial models (e.g., Frazier and Clifton, 1996; Friederici, 2002) assign a module-specific functional interpretation to ERP components and postulate that syntactic processing interacts with other linguistic information only at the output level. These models have been challenged by findings that ERP effects vary in their presence, latency, amplitude, and scalp distribution depending on the morphosyntactic elements in question (for details see Dube et al., 2016). Alternatively, non-modular interactive models assume that during auditory sentence processing all sources of information exert a non-hierarchical direct and parallel influence (MacDonald et al., 1994; van den Brink and Hagoort, 2004; Pickering and Garrod, 2013). Specifically, van den Brink and Hagoort (2004), suggest that when the word category is encapsulated in the suffix or prefix of a word, semantic processing is postponed until the word has been completely heard and the word category has become available. Finally, the extended Argument Dependency Model (Bornkessel-Schlesewsky and Schlesewsky, 2009), incorporates aspects of serial and parallel processing and assumes continuous flow of information between processing stages. This may lead to temporal overlap between stages and represents an “integrating window” which allows reciprocal influences between different types of information (Mancini, 2018).

We compared verbal agreement processing in Farsi as a native language and a proficient second language (L1 vs. L2), which has rarely been addressed. English materials was presented to Chinese and Japanese bilinguals by Chen et al. (2007) and Ojima et al. (2005), respectively, and Rossi et al. (2006) presented German sentences to Italian bilinguals. Consistently, verbal agreement violations elicited a LAN (around 300–500 ms) in (late) L2 learners but the presence of a P600 varied. Hence, relative to native speakers, late L2 learners may use different neural mechanisms to process verbal agreement. Some researchers argue that morphosyntactic real-time processing in late L2 learners depends on proficiency rather than age of acquisition (AoA) (Steinhauer et al., 2009). Similarly, Hernandez (2013) proposed that academic proficiency sharpens brain areas involved in language processing, whether the language is learned during childhood or later. Therefore, native-like syntax processing may be achieved at high proficiency even in late-acquired L2.

Sentence final subject-verb agreement processing requires the integration of grammatical information across linguistic units of the preceding sentence, which have to be held activated in working memory (Badecker and Kuminiak, 2007). Due to the limited capacity of working memory, also selective attention as another executive function (Zink et al., 2021) is required to focus on relevant aspects of the task (Ku, 2018), explaining enhanced attentional control in bilinguals (Ouzia et al., 2019). Therefore, we assessed the involvement of language-related working memory capacity in grammaticality processing and its neural correlates.

To address our objectives we employed highly competent L1 and L2 speakers of Farsi in an auditory ERP paradigm featuring morphosyntactic violations in Farsi sentences. In line with the SOV structure of Farsi, morphosyntactic processing was manipulated at sentence-final positions. Although sentence-final positions are commonly avoided in neurolinguistic studies in order to exclude sentence wrap-up processes, this strategy has come under criticism (Stowe et al., 2018). Independent of syntactic or semantic correctness, global ERP shifts have been observed at the end of “strings,” that is, a constituent at any position in a sentence, whether sentence final or mid-sentence (Shen et al., 2013).

We expected LAN and P600 effects in response to subject-verb agreements. Since this is the first ERP study of a spoken SOV nominative-accusative language, the distribution and timing of these components was of particular interest. Since even proficient L2 speakers have to invest more effort than L1 speakers in morphosyntactic processing, they might show larger or later LAN or P600 components (Clahsen and Felser, 2006; Kotz, 2008; Steinhauer et al., 2009; Caffarra et al., 2015), resembling similar effects in semantic processes (Hohlfeld et al., 2004). Finally, we expected that the P600 amplitude, which may be a signal of more controlled and effortful processing, might relate to working memory capacity, especially in nonnative speakers.

## Methods

### Participants

We recruited 69 healthy, normally hearing adults among Iranian students. After excluding eight participants due to EEG artifacts, the final sample consisted in 28 native Farsi speakers and 33 native Turkish speakers who had acquired Farsi during elementary school from age seven (see Table 1 for demographic details). L2 speakers had Turkish parents and grown up in Turkish provinces of Iran (Tabriz, Urmia, Ardabil, and Zanjan); they reported speaking Turkish at home and with their families but had received their formal education in Farsi, had spent at least 5 years in a Farsi-speaking city (range 5–7 years) and used both languages in their daily activities.

**TABLE 1:** Demographic characteristics and language proficiency.

	Accuracy					
	Native speakers (*N* = 28, 17 F)		Non-native speakers (*N* = 33, 15 F)		*t-*Test*	
	M (SD)	Range	M (SD)	Range	*t (59)*	*P*
Age	26.82 (3.878)	23–38	26.52 (3.03)	23–34	0.346	0.731
Speaking Pro.	5.50 (.509)	5–6	5.52 (0.755)	4–6	−0.090	0.928
Listening Pro.	6 (0)	6–6	6 (0)	6–6	−	−
VWM	76.52 (7.30)	57.4–88.9	76.93 (8.13)	63–90.7	−0.209	0.835
SES (P. edu.)	4.82 (1.39)	1–7	4.15 (1.60)	1–7	1.728	0.089
SES (P. occ.)	5.96 (2.0)	3–9	6.12 (1.86)	3–9	−0.316	0.753
Y. of Edu.	18.97 (1.87)	17–22.6	19.02 (1.59)	17–21.6	−0.103	0.919

*F, Female; Pro., Proficiency; VWM, verbal working memory; SES, socioeconomic status; P. edu, Parental education; P. occ, Parental occupation; Y. of Edu, years of education.*

*Independent samples test.

In the absence of standardized language proficiency tests in Farsi, proficiency was determined according to several criteria: language-learning, self-rating of speaking and listening proficiency, and a structured interview in Farsi. Based on these measures, participants in both groups were highly proficient in Farsi. According to parental education and occupation, socioeconomic status was similar (Hollingshead, 1975). Participants were required to be right-handed (Oldfield, 1971), assessed for working memory capacity with a Reading Span Test (Khodadadi et al., 2014); they provided written informed consent and were reimbursed. The study was approved by the Research Ethical Committee of Iran University of Medical Sciences (IR.IUMS.REC.1398.465).

### Stimuli

Stimulus materials consisted in 120 Farsi sentences, half of which were syntactically correct, whereas the others included a morphosyntactic subject-verb agreement violation (for examples, see Table 2). Further 120 sentences were derived by converting the correct and incorrect sentences of the first list into their incorrect and correct counterparts, respectively. Each list was assigned to half of the participants (per group). To be noted, this procedure does not provide a control that the same syllables are morphosyntactically correct and incorrect (in different sentences). Hence, all sentences were presented in their correct and incorrect versions (albeit to different participants) and no sentence (whether correct or incorrect) was presented twice to the same participant.

**TABLE 2:** Examples for sentence materials and descriptive statistics of the dependent measures.

Example in Farsi with Transliteration and Literal Translations in Parenthesis*				
**Correct**		**Incorrect**		

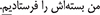		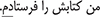		
Man baste-ʔ-aš râ ferestâdim. I parcel-HI-OBJ-CLT.Def send.PAST-1PL (I sent her parcel.)		Man ketab-aš râ ferestâdam. I book-BBJ-CLT.Def send.PAST-1SG (I sent her book.)		

**Mean durations of audio stimuli**				

	**Correct (SD)**	**Incorrect (SD)**	***t*-test (df = 118)**	

Target-syllable (ms)	468.9 (90.7)	480.15 (87.1)	*t* = −0.69	*p* = 0.49
Pre-target auditory signal (s)	2.73 (0.15)	2.78 (0.18)	*t* = −1.81	*p* = 0.07
First consonant (ms)	33.13 (18.24)	33.75 (19.23)	*t* = −0.18	*p* = 0.86
Vowels (ms)	184.1 (20.5)	192.1 (23.2)	*t* = −2.01	*p* = 0.047
Post-vowels (ms)	249.05 (76.0)	257.0 (83.1)	*t* = −0.55	*p* = 0.59

*The critical syllable is underlined.

*1, First person; SG, Singular; PAST, Past; 3, Third person; PL, Plural; HI, Hiatus; OBJ-CLT, Objective clitic; Def, Definitive, Sentence duration > 3.24 s.*

All sentences followed the structure Subject + Object + Verb. Verbs were regular and highly frequent (Anvari and Givi, 2006). There are no norms for syllable frequency in Farsi but also incorrect syllables could be part of correct words (in other contexts). Sentences included only past tense transitive verbs of a similar kind of transitivity (direct); no copula verbs were used. Half the sentences in each language were morphosyntactically correct, whereas the other half included agreement violations in the verb.

The sentences were pronounced by a female speaker in natural tempo and prosody, recorded with a Sony ICD-UX560 digital voice recorder at 16-bit resolution and 44.1 KHz sampling rate, and edited with WavePad audio editing software. The onsets of the target syllable (the last syllable of the sentence final word) were identified and coded by two trained coders using auditory cues and visual inspection of sound spectrograms.

### Procedure

The behavioral and EEG sessions were conducted at the linguistics department of Tarbiat Modares University and National Brain Mapping Lab, taking 120 and 70 min, respectively. During the behavioral session, participants performed the Reading Span Test and were assessed for socioeconomic status, handedness and language proficiency. In the EEG session participants sat in an acoustically and electrically shielded booth. After applying the electrode cap, sentences were presented *via* earphones, controlled by MATLAB’s Psychtoolbox, intermixing grammatical and ungrammatical sentences in a fixed pseudo randomized order where none of the two different conditions appeared more than twice in a row.

A black central cross as fixation cue was displayed on a gray monitor 70 cm away from the participant, shown throughout sentence presentation, serving also as a cue to refrain from blinking and eye movements. Sentence presentation started together with the fixation cross and was followed by an auditory prompt to make a grammaticality judgment with one of two keys on the keyboard. Two seconds after the response the next trial started.

### Data Acquisition and Preprocessing

EEG was sampled at 512 Hz from a 64-channel g.HIamp and international 10–10 electrode configuration, using active electrodes and right mastoid as online reference and Fpz as ground. Horizontal eye movements were monitored with electrodes at the outer canthi; vertical eye movements were monitored with a right infraocular electrode and Fp2. Offline EEG preprocessing and analyses were performed in MATLAB^®^ R2016b software and the EEGLAB toolbox (Delorme and Makeig, 2004). Data were band-pass filtered at 0.1 to 40 Hz. Artifact contaminated channels were rejected automatically and based on visual inspection; EEG channels were re-calculated to average reference. Ocular artifacts were corrected using the automatic artifact removal (AAR) toolbox of EEGLAB, followed by independent component analysis using the runica routine of EEGLAB. Bad channels were reconstructed by interpolating weighted averages of neighboring channels by spherical spline interpolation. Continuous data were segmented into 1,600-ms epochs starting 100 ms before the onset of the critical (final) syllables, which on average lasted for 474 ms (±97). Epochs with a voltage range exceeding ± 100 μV at any channel were rejected. Only trials in which participants responded correctly were averaged. Thus, 11.5% of trials were removed. ERPs were computed separately for stimulus type and electrode site. There were no significant differences in the number of rejected trials between conditions in the Farsi or Turkish group [ts(59) = −0.231and −0.235, ps = 0.818 and 0.815, respectively]. Moreover, there were no significant differences in the number of grammatical or ungrammatical rejected trials in the Farsi or Turkish group [*t*(59) = −1.088 and −0.866, *p* = 0.281 and 0.390, respectively].

### Data Analysis

For a suitable synchronization point and baseline, we considered that the pattern of all target syllables was CVC(C), with the first consonant being matched for correct and incorrect conditions; hence, only the nucleus of the last syllable differed. Because the mean duration of the first consonant was approximately 33 ms, we used a 100-ms baseline from −65 to +35 ms relative to syllable onset. Visual inspection of waveforms and topographical maps appeared to show effects of grammatically, possibly reflecting LAN- and P600-like components. Amplitudes were analyzed for an anterior region of interest (ROI) at electrode sites F1, F2, F3, F4, FC1, FC2, FC3, FC4, Fz, and FCz, and a posterior ROI comprising electrodes P1, P2, P3, P4, P5, P6, PO3, PO4, O1, O2, Pz, POz, and Oz. Within each ROI amplitudes were averaged across the left, midline and right electrodes and for the following intervals: 35–135 ms, examining effects of phonological features; 300–500 (LAN), 500–700 (P600) and 700–1,100 ms. The latter interval has been shown to be sensitive to L1/L2 differences. Analyses of variance (ANOVAs) included group factor AoA (native vs. nonnative speakers) and repeated measures on congruency (canonical, violation) and laterality (left, midline, right).

## Results

### Behavioral Results

In the grammaticality judgment task, the percentage of correct responses was very high and similar in native (*M* = 98.74, ± 1.73) and nonnative (*M* = 99.03, ± 1.32) speakers [*t*(59) = −0.74, *p* = 0.463]. Also, the groups did not differ in reaction times [*t*(59) = 0.96, *p* = 0.34) (*M* = 0.70 s ± 0.33 vs. *M* = 0.63 s ± 0.24).

### Electrophysiological Results

**FIGURE 1:**
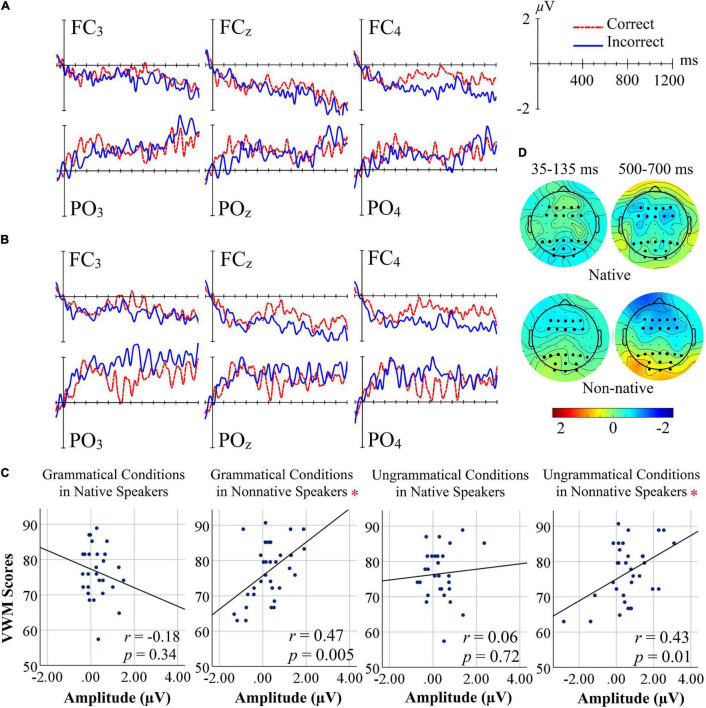
**(A)** Grand-average ERPs for incorrect versus correct conditions in the anterior and posterior region of interest of Native speakers. **(B)** Grand-average ERPs for incorrect versus correct conditions in the anterior and posterior region of interest of Non-native speakers. **(C)** Scatter plots showing the relationship between VWM and mean amplitude at posterior ROIs (500–700 ms) in native and nonnative speakers per condition. Significant correlations are marked with a red asterisk. **(D)** Grand-average difference topographies for incorrect minus correct conditions for all electrodes during the 35–135, and 535–635 ms intervals. Bigger dots indicate the ROI electrodes.

Figures 1A,B presents ERPs at representative electrodes and Figure 1D shows topographical maps of the grammaticality effect. ANOVA of ERP amplitudes in the 35–135 ms interval revealed a significant interaction between Grammaticality and AoA for the anterior ROI [*F*(1, 59) = 4.31, *p* = 0.042, $\eta_{\text{p}}^{2}$ 0.068]. *Post hoc* paired samples *t*-tests showed a significant effect of Grammaticality only in non-native [*t*(32) = −2.21, *p* = 0.034] but not in native speakers [*t*(27) = 0.870, *p* = 0.392]. Also, the posterior ROI revealed a Grammaticality x AoA interaction [*F*(1, 59) = 4.64, *p* = 0.035, $\eta_{\text{p}}^{2}$ 0.073]. *Post hoc t*-tests showed no condition differences in native or non-native speakers and no group difference in the grammatical condition, but larger amplitudes in nonnative than native speakers in the ungrammatical condition [*t*(32) = −2.76, *p* = 0.008]. In the LAN interval (300–500 ms) there were no significant effects (Fs < 1).

The 500–700-ms interval revealed a main effect of Grammaticality [*F*(1, 59) = 4.72, *p* = 0.034, $\eta_{\text{p}}^{2}$ 0.074], in the posterior ROI with more positive amplitudes to incorrect than correct sentences. Although Figure 1 appears to indicate a stronger grammaticality effect in non-native speakers, this could not be statistically verified (*F* < 2). Based on the visual impression of Figure 1 we conducted an exploratory analysis of the anterior ROI in the 500–700 ms interval. Here, main effects of Laterality [*F*(1, 59) = 6.20, *p* = 0.003, $\eta_{\text{p}}^{2}$ 0.095] and Grammaticality [*F*(1, 59) = 7.51, *p* = 0.008, $\eta_{\text{p}}^{2}$ 0.113] were significant, with more negative amplitudes to incorrect than correct sentences. Again the interaction of Grammaticality and AoA was not significant. *Post hoc* analysis of laterality indicated smaller amplitudes in left as compared to right electrodes [*t*(60) = 3.01, *p* = 0.004], and compared to midline electrodes [*t*(60) = 3.31, *p* = 0.002], but no difference between midline versus right electrodes [*t*(60) = −0.579, *p* = 0.565]. The final interval (700–1,100 ms) yielded no significant effects (Fs < 1) (see Supplementary Tables 1, 2).

Mean reading span performance was indistinguishable for native (*M* = 76.52 ± 7.30) and non-native speakers (*M* = 76.93 ± 8.13) [*t*(59) = −0.21, *p* = 0.84]. However, reading span correlated positively with performance accuracy in nonnative speakers (*r* = 0.396, *p* = 0.022) but not in native speakers (*r* = −0.023, *p* = 0.906); the difference of these correlations was a strong trend (*z* = −1.632; *p* = 0.051). A scatter plot for this relationship is presented in Supplementary Figure 1, showing a strong effect for accuracy with combination of some outliers in nonnative speakers. Also, in nonnative speakers reading span correlated with ERP amplitudes in the posterior ROI in the 500–700-ms interval in both grammatical (*r* = 0.474, *p* = 0.005) and ungrammatical (*r* = 0.435, *p* = 0.011) conditions, whereas in native speakers both correlations failed significance (ps = 0.340 and 0.727) (Figure 1C); both correlations were significantly different between groups (*z* = −2.601 and −1.977; *p* = 0.005 and 0.024).

Because the grammatically effect in the 35--135-ms interval might relate to condition differences in the acoustic properties of correct and incorrect auditory stimuli we used PRAAT 6.0.49^1^ to compute the mean average of the acoustic features of vowels across conditions during the 35–135-ms interval (Table 3). Loudness (dB), pitch (Hz), jitter (ms), shimmer (dB), and the harmonic-to-noise ratio (HRN, dB), were indistinct; however, there were significant condition differences in the first formant [*t*(114) = 2.136, *p* = 0.035] and second formant [*t*(114) = −2.658, *p* = 0.009] (Supplementary Table 3).

**TABLE 3:** Values depict mean and standard deviation of the acoustic proprieties of stimuli.

	Grammatical condition		Ungrammatical condition			
	Mean	SD	Mean	SD	*t*	*P*
Loudness (dB)	73.25	3.88	72.48	3.46	1.124	0.263
Pitch F0 (Hz)	187.74	10.85	191.18	9.73	–1.799	0.075
Jitter (μs)	45.01	26.26	44.26	30.60	0.141	0.888
Shimmer (dB)	0.737	0.320	0.644	0.268	1.712	0.090
HNR (dB)	10.73	3.68	13.97	23.71	–1.018	0.311
F1	691.55	248.16	595.74	234.95	2.136	0.035
F2	1,958.76	460.34	2,205.54	535.44	–2.658	0.009

*t-values and P-values show the result of the Independent sample t-test tests used to statistically compare the acoustic proprieties across grammatical and ungrammatical conditions.*

To explore the relationship between the anterior and posterior ERP effects of grammaticality in the 500–700-ms interval we correlated them across participants and found the right anterior and left posterior effects to be strongly correlated (*r* = −0.731, *p* < 0.0001) as were the right posterior and left anterior effects (*r* = −0.694, *p* < 0.0001).

## Discussion

We investigated—to the best of our knowledge for the first time—ERP responses to auditory suffix-sized targets in a canonical SOV nominative-accusative language (Farsi), recorded in L1 speakers and highly proficient late L2 learners, who achieved indistinguishably high performance. In ERPs, verbal agreement violations elicited a very early anterior negativity (specifically in non-native speakers) followed by late posterior positive grammaticality effects, reminiscent of the P600. An exploratory analysis also showed an anterior negative-going grammaticality effect in the 500–700-ms interval.

Very early ERP effects of syntactic manipulations are primarily associated with the auditory modality. Early negativities to subject-verb agreement violations with onsets around 150 and 100 ms, respectively, have been found—controlling for acoustic confounds with cross-splicing—by Shen et al. (2013) and Hasting and Kotz (2008), supporting an account of the present effects in terms of grammaticality. These previous studies mainly differed from ours by positioning the S-V agreement violations at mid-sentence, which may explain the slightly different outcomes. Alternatively, our early grammaticality effects may be attributed to acoustic differences in F1 and F2 formants. F1 was higher in grammatical than ungrammatical conditions, and the reverse held for F2, reflecting acoustic characteristics of the relatively predominance of vowels /i/ and /a/ in these conditions, respectively. The increased early anterior negativity in the non-native speakers resembles the N100 component, a prominent waveform deflection between 90 and 160 ms (Obleser et al., 2003), suggested to reflect the discrimination of phonemes and auditory categories (Friederici, 2011). This view is supported by neuroimaging studies on phoneme processing localizing the N100 component for vowels and consonants in Heschl’s gyrus and planum temporale (Obleser et al., 2003; Friederici, 2017). In addition, Obleser et al. (2003) suggested that the spatial mapping of N100 is dominated by place information from vowels. In other words, different locations of N100 along the posterior-anterior axis depend on the place of vowel articulation but are independent of syllable onsets. Therefore, it can be argued that neural responses to the front vowels /a/ and /i/ (pronounced with the highest part of the tongue positioned in front of the mouth), as used here, may generate our N100 at anterior scalp.

Interestingly, the early effects were present in non-native but not detectable in native speakers. Therefore, non-native speakers may be more sensitive to the phonological characteristics of syllables in grammatical and ungrammatical conditions. This may reflect the difference in formant frequencies in Farsi and Turkish vowels (Modaresi Ghavami, 2011; Korkmaz and Boyaci, 2018) where frequencies of F1 and F2 for the Farsi vowels /a/ and /i/ are higher than in their Turkish counterparts. Since we are not aware of similar reports, this finding should be followed up in future studies.

In the 500–700-ms interval we found late effects of grammaticality at both anterior and posterior sites. These late effects may reflect the targeting of rules for interpretation, engaged in the mapping between morphosyntactic and thematic information (Mancini, 2018). In other words, after all available information has been processed, reanalysis may be triggered in order to arrive at a coherent sentence interpretation. The posterior positive late effect of grammaticality was accompanied by an anterior negativity. This anterior-posterior bipolar scalp topography appears to be at variance with the commonly seen centro-parietal P600. However, bipolar P600 distributions have been reported in response to morphosyntax-manipulated spoken German sentences if the probability of sentence incorrectness was at 50% (Xu et al., 2019, 2021b), whereas more infrequent errors elicited a typical centro-parietal P600 (Xu et al., 2021b). Therefore, our late grammaticality effect resembles previous findings in a language with flexible word order (German).

What is the significance of the anterior effect? A first point to be considered, is the substantial correlation of the anterior and posterior grammaticality effects, arguing for a unitary generator or system of highly coupled generators, producing a bipolar anterior negative/posterior positive topography (Luck, 2014). In a system of generators, one of them might be responsible for the posterior positivity while the other may produce anterior, LAN-like activities. This speculation implies a late LAN-like activity, as a consequence of encoding all morphosyntactic information in the same suffix, required in Farsi. The idea of parallel, highly coupled LAN-like and P600-like processes aligns with the extended argument dependency model (Bornkessel and Schlesewsky, 2006), which assumes a cascaded organization of the linguistic form-to-meaning mapping and a hierarchy of processing stages in which the analysis of syntactic information dominates other types of analysis, based on a cascaded, continuous flow model of information between processing stages (McClelland, 1979; Mancini, 2018).

Interestingly, specifically in nonnative speakers reading span performance correlated positively with both, performance accuracy in the grammaticality judgment task, as well as with the late posterior positivity. These findings indicate the involvement of working memory resources to establish the dependency between the verb and its argument, which seems to be more important for nonnative speakers, where grammatically judgments may be less automatic than in native speakers and require the recruitment of executive functions, such as working memory and selective attention (Ku, 2018). This perspective on the late positivity as being related to executive functions, is in line with suggestions of Xu et al. (2019, 2021a,2021b), who observed P600 attenuations when proactive recruitments of cognitive resources was likely and with the task dependence of P600 reported by Schacht et al. (2014).

Regardless of the typological characteristics of nominative/ergative case-marking of subjects, the presence of a P600 effect in the current study is consistent with existing findings in native speakers of SOV languages of ergative-absolutive Basque (Zawiszewski and Friederici, 2009; Dıaz et al., 2011; Zawiszewski et al., 2016; Chow et al., 2018) and split-ergative Hindi (Nevins et al., 2007). It is noteworthy to mention that no indication of P600 was found in nominative-marked cases in Hindi speakers (Choudhary et al., 2009) during the double case processing (ergative vs. nominative) and a more pronounced P600 was observed during the dative-marked cases as opposed to nominative- and accusative-marked cases in German (Frisch and Schlesewsky, 2005), reflecting the sensitivity of the brain either to linguistic distinctions between different cases or to non-default cases (i.e., ergative in Hindi or dative in German). With all caution (there was no significant interaction), if replicable, the numerically larger P600 in non-native than in native speakers could be partially attributed to the involvement of higher cognitive control in second language processing, which was indicated also for an SOV language in our recent fMRI study (Meykadeh et al., 2021). Furthermore, the observation of an early negativity with shorter latency than the classical N400 (100–300 ms for Hindi; Choudhary et al., 2009) and more posterior scalp distribution than the classical LAN (for Basque; Zawiszewski and Friederici, 2009; Dıaz et al., 2011; Zawiszewski et al., 2016; Chow et al., 2018) in SOV languages may be a consequence of the ergativity-marked case used.

Independent of syntactic correctness, we observed a bipolar anterior string-final negativity and posterior string-final positivity at the end of the recording epoch. Similar shifts were reported at mid-positions of spoken sentences of SVO languages (Hasting and Kotz, 2008; Shen et al., 2013). Taken together, these observations undermine the status of negative/positive effects at sentence-final positions as a sign of sentence wrap-up. Since posterior string-final positivities have been reported also in written sentences (Osterhout and Mobley, 1995; Palolahti et al., 2005; Silva-Pereyra and Carreiras, 2007; Kos et al., 2010), at least these effects seem to be modality-independent. Accordingly, the string final effects in our data may reflect on-line processing of the final sentence constituent. Further, the view that sentence-level structure building is delayed until the end of the clause, has been mostly rejected based on the rapid sensitivity to the semantic properties of the current word (e.g., DeLong et al., 2005; Van Berkum et al., 2005) and its syntactic properties (e.g., Friederici, 2002; Kaan and Swaab, 2002).

In conclusion, we present first ERP evidence of morphosyntactic violations in the final verb in spoken sentences of a SOV language (Farsi). L2 speakers who were indistinguishable in their language competence at the performance level, showed subtle differences in early ERPs, pointing at differential sensitivity to acoustic properties. Given the near-absence of neuroscientific studies of SOV languages, which is the dominant word order in the worlds languages (Dryer, 2005), the present report and our companion fMRI paper (Meykadeh et al., 2021) represents an important entry point into a understudied field of neurolinguistic exploration.

## Data Availability Statement

The datasets presented in this article are not readily available because participants did not provide permission for data to be shared publicly. Requests to access the datasets should be directed to SM, a.meykadeh@modares.ac.ir.

## Ethics Statement

The studies involving human participants were reviewed and approved by Research Ethical Committee of Iran University of Medical Sciences (IR.IUMS.REC.1398.465). The patients/participants provided their written informed consent to participate in this study.

## Author Contributions

SM planned the study, collected and analyzed the data, and wrote the manuscript. AG supervised the work and provided valuable laboratory resources. AN contributed to designing the task. HA provided the essential resources. WS contributed to the planning of the work, supervised data analysis, and edited the manuscript. All authors contributed to the article and approved the submitted version.

## Conflict of Interest

The authors declare that the research was conducted in the absence of any commercial or financial relationships that could be construed as a potential conflict of interest.

## Publisher’s Note

All claims expressed in this article are solely those of the authors and do not necessarily represent those of their affiliated organizations, or those of the publisher, the editors and the reviewers. Any product that may be evaluated in this article, or claim that may be made by its manufacturer, is not guaranteed or endorsed by the publisher.

## Footnotes

1
http://www.praat.org
