# Supplementary material for: First Event-Related Potentials Evidence of Auditory Morphosyntactic Processing in a Subject-Object-Verb Nominative-Accusative Language (Farsi)
Source: Front Psychol. 2021 Dec 16;12:698165. doi: 10.3389/fpsyg.2021.698165 (PMC8716833; doi:10.3389/fpsyg.2021.698165)
Supplement: Supplementary file 1 [file Data_Sheet_1.pdf]

Supplementary Material

Supplementary Table 1. Results of repeated-measures ANOVAs, for posterior and anterior regions of interest in the intervals 35-135, 300-500, 500-700, and 700-1100 ms.

	35 – 135 ms			35 – 135 ms			300 – 500 ms			500 – 700 ms			700 – 1100 ms			500 – 700 ms		
	Anterior			Posterior			Anterior			Posterior			Posterior			Anterior (Exploratory)		
	F	p	η_p^2	F	p	η_p^2	F	p	η_p^2	F	p	η_p^2	F	p	η_p^2	F	p	η_p^2
G	0.47	0.49	0.008	0.22	0.64	0.004	2.29	0.136	0.037	4.72	0.03	0.074	0.73	0.39	0.012	7.51	0.008	0.113
G x AoA	4.31	0.04	0.068	4.64	0.03	0.073	0.06	0.811	0.001	1.94	0.17	0.032	0.10	0.75	0.002	0.37	0.544	0.006
L	2.07	0.13	0.034	1.62	0.20	0.027	8.90	0.000	0.131	1.72	0.18	0.028	2.75	0.07	0.045	6.21	0.003	0.095
L x AoA	0.59	0.55	0.010	0.12	0.88	0.002	0.05	0.953	0.001	0.19	0.83	0.003	0.03	0.97	0.001	0.19	0.889	0.002
G x L	0.17	0.85	0.003	0.95	0.39	0.016	0.44	0.646	0.007	1.36	0.26	0.023	0.63	0.53	0.011	0.41	0.664	0.007
G x L x AoA	0.18	0.83	0.003	0.96	0.39	0.016	0.01	0.990	0.000	0.23	0.79	0.004	0.52	0.59	0.009	0.57	0.569	0.010

G = Grammaticality, L = Laterality, AoA = Age of Acquisition

Supplementary Table 2. Results of post-hoc analyses for effect of factors Grammaticality and Laterality using Paired Samples *t*-test, in the intervals 35-135 and 500-700 ms.

			Mean	Standard Deviation	Standard Error of the Mean	95% Confidence Interval of the Difference		*t*	df	Sign. (2-tailed)
						Lower	Upper			
Grammaticality 35 – 135 ms	Ant.	Native	0.090	0.54	0.103	-0.122	0.302	0.0870	27	0.392
		Non-native	-0.179	0.46	0.081	-0.344	-0.014	-2.213	32	0.034
	Post.	Native	-0.126	0.57	0.108	-0.349	0.096	-1.164	27	0.255
		Non-native	0.198	0.59	0.103	-0.013	0.409	1.910	32	0.065
Laterality 500 – 700 ms (Anterior ROI)	Left vs. Right		0.154	0.40	0.051	0.051	0.256	3.01	60	0.004
	Left vs. Midline		0.189	0.44	0.057	0.075	0.303	3.32	60	0.002
	Right vs. Midline		-0.034	0.47	0.060	-0.155	0.085	-0.579	60	0.565

Ant. = Anterior, Post. = Posterior, Native = Native Speakers, Non-native = Non-Native Speakers

Supplementary Table 3. Results of independent samples *t*-test) of group differences in the reading span task and condition differences in the acoustic properties of the critical syllables.

	Mean difference	Standard Error difference	95% Confidence Interval of the Difference		*t*	df	Sign. (2-tailed)
			Lower	Upper			
WM	-0.415	1.995	-4.410	3.574	-0.21	59	0.84
F1	95.809	44.858	6.944	184.673	2.136	114	0.035
F2	-246.77	92.855	-430.720	-62.829	-2.658	114	0.009

WM = Working memory, F1 = Formant 1, F2 = Formant 2

Supplementary Figure 1. Scatter plot showing the correlation between response accuracy and VWM in native and nonnative speakers.

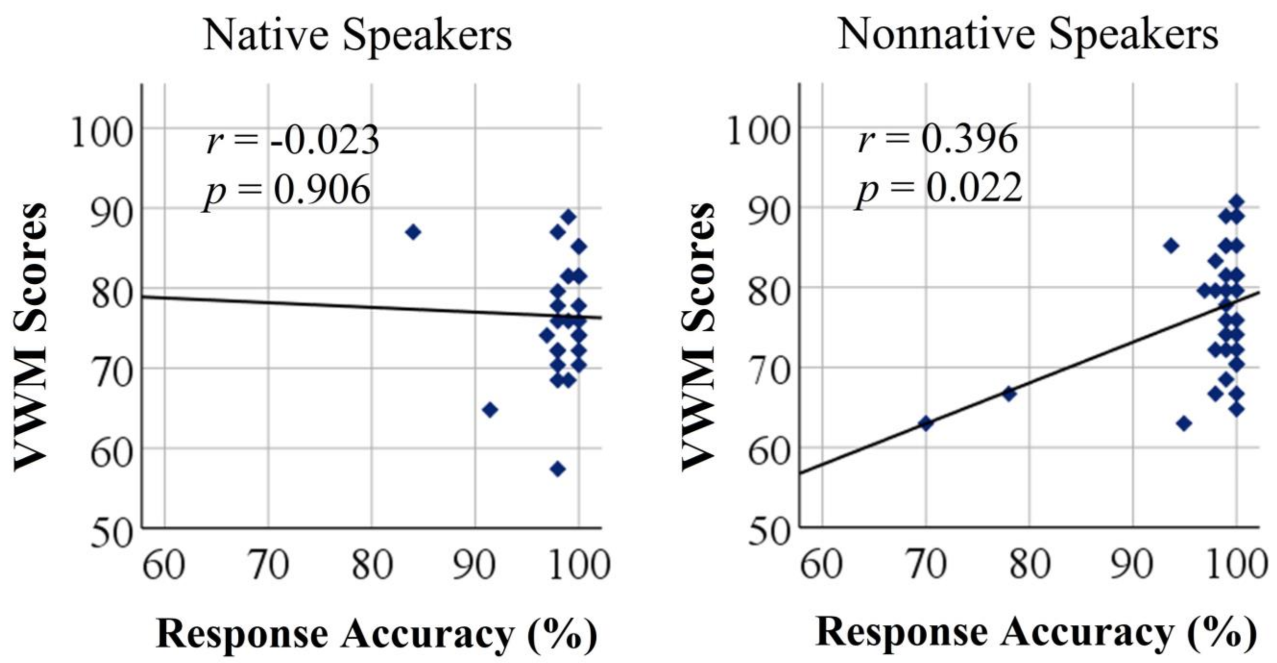

frontiers

frontiers
